# Socioeconomic Disparities in Health Care Consumption: Using the 2018-China Family Panel Studies

**DOI:** 10.3390/ijerph19127359

**Published:** 2022-06-15

**Authors:** Enkai Guo, Huamei Zhong, Yang Gao, Jing Li, Zhaohong Wang

**Affiliations:** 1 College of P.E and Sports, Beijing Normal University, Beijing 100875, China; guoenkai18@126.com (E.G.); 201931070006@mail.bnu.edu.cn (Y.G.); sun14170045@163.com (J.L.); 2 School of Physical Education and Sports Science, Fujian Normal University, Fuzhou 350117, China; zhonghua6266@126.com

**Keywords:** socioeconomic status, physical exercise, health care consumption, probit model, Tobit model, mediation effect

## Abstract

The existing research on residents’ health care consumption mostly covers medical care consumption and seldom regards residents’ health care consumption as an independent research object. This article takes residents’ healthcare consumption as the research object and aims to explore the impact of socioeconomic status on healthcare consumption and its mechanisms. The data of this study came from the 2018-China Family Panel Studies (CFPS). The binary probit regression model and the Tobit model explored the impact mechanism of residents’ income, education, occupation, and physical activity on health care consumption decision-making and health care expenditure, respectively. The research results showed that, from the perspective of the direct influence mechanism, residents’ work income (0.029, *p* < 0.01) and education level (811.149, *p* < 0.01) had a significant positive impact on health care consumption. Residents whose occupations (−99.697, *p* < 0.01) tend to be more skilled and also have higher health care consumption. From the perspective of the mediating mechanism, residents’ physical exercise duration had a significant positive impact on their participation in healthcare consumption (0.005, *p* < 0.01) but had a weaker impact on healthcare consumption expenditure (21.678, *p* < 0.1). In general, socioeconomic status represented by income, education, and occupation had a significant positive impact on residents’ health care consumption. The duration of physical exercise also played an important mediating role.

## 1. Introduction

Globally, more countries and regions continue to promote health care consumption and improve the health of residents by implementing regional policies and measures related to national fitness. Existing studies have shown that health care consumption in various countries has grown significantly, especially in developing countries represented by China [1]. According to data from the National Bureau of Statistics of China, in 2018, 2019, and 2021, China’s per capita health care consumption was 1902 yuan, 1843 yuan, and 2115 yuan, respectively. Consumption. In 2021, the per capita health care consumption expenditure will account for 8.8% of the per capita consumption expenditure in the same period, and the growth rate will be 1.2 percentage points higher than the per capita consumption expenditure of national residents [2]. It can be seen that in 2021, with the increase in health care consumption demands of residents of all socioeconomic statuses, health care consumption decisions and health care consumption expenditures will also be positively affected [3]. More importantly, socioeconomic status, including work income, education, occupation, and other factors, plays a role in residents’ health care consumption [4].

In the existing research, scholars usually divide the socioeconomic status of residents according to indicators, such as personal income, education, social prestige, and occupational professionalism. Residents with a low income, few years of education, low social prestige, or labor-type occupations are often classified as having a low socioeconomic status [5,6]. Residents with a high income, high education, high social prestige, or skilled jobs are classified as having a higher socioeconomic status. In this study, we covered socioeconomic status similar to the existing literature. That is, socioeconomic status refers to an overall measure of a person’s socioeconomic status relative to other people’s factors such as income, education, and occupation. Moreover, residents with different socioeconomic statuses often have different needs for health care consumption. For example, Yin et al. (2007) pointed out that income level is an important factor affecting residents’ health care consumption. Among urban residents, the residents of the low-income class have a higher marginal propensity to consume medical care, while the residents of the high-income class have a lower demand for medical care consumption, and the consumption expenditure of the low-income class has increased significantly [7]. Yao (2020) found that age and education level also have an impact on health care consumption, among which the social health care consumption demand and expenditure of the elderly group and the group with a higher education investment level are higher [8]. Numerous studies have found that there is a positive relationship between socioeconomic status and residents’ health care behavior [9,10,11]. This relationship is reasonable because residents with higher economic income, more years of education, and skilled jobs pay more attention to physical health and invest more in health capital to maintain a higher quality of life. Therefore, existing studies have found that income, education, and occupation are important indicators for measuring the socioeconomic status of residents. Combined with the data types of CFPS, this study also chooses to examine the socioeconomic status of residents from three aspects of income, education, and occupation and explore the relationship between these three indicators and health care consumption. Based on this, the following research hypotheses are proposed:

**Hypothesis** **1** **(H1).** *Residents’ work income is significantly positively correlated with health care consumption expenditures.*

**Hypothesis** **2** **(H2).** *The education level of residents has a significant positive correlation with health care consumption expenditures*.

**Hypothesis** **3** **(H3).** *Residents with skilled occupations have higher spending on health care.*

However, few studies have explored the underlying mechanism of the relationship between socioeconomic status and residents’ health care consumption. Therefore, the purpose of this study is to fully explore the relationship and mechanism between socioeconomic status and residents’ health care consumption. This study is carried out at the individual level, so the influence of individual lifestyle and behavior on health care consumption is inevitable. Wang (2017) used five indicators of smoking, drinking, exercise, rest, and relaxation, and routine physical examination to measure residents’ lifestyles. It was found that social members with higher education and economic levels pay more attention to physical health, and their lifestyles gradually change from unhealthy to healthy. Furthermore, the enthusiasm of residents to participate in health care consumption is relatively high. On the other hand, the lifestyle of lower socioeconomic status members has changed from healthy to mixed; that is, they are more restricted and less likely to participate in health care consumption [12]. Other scholars took Beijing–Tianjin–Hebei urban residents as research objects and found that physical exercise has a significantly higher impact on residents’ health than health care consumption; that is, physical exercise behavior better measures residents’ health [13]. It can be found from the above that residents’ physical exercise behavior better represents the health status of residents, and health status is an important factor affecting residents’ health care consumption. Therefore, this paper selects the duration of residents’ participation in physical exercise as an intermediary variable to explore its mediating effect on the relationship between residents’ socioeconomic status and health care consumption. The specific research hypotheses are as follows:

**Hypothesis** **4** **(H4).** *Residents’ exercise duration has a significant positive correlation with health care consumption expenditure.*

**Hypothesis** **5** **(H5).** *Residents’ exercise duration has a mediating effect on the influence of socioeconomic status and health care consumption.*

This study took residents’ health care consumption, including the consumption of purchasing fitness equipment, health products, participating in exercise, etc., as the research object to explore the effect of education, income, occupation, and other socioeconomic status factors on residents’ health care consumption expenditure. In addition, this study also started from the potential mechanism, introduced physical exercise variables, and explored its mediating effect.

## 2. Methods

### 2.1. Data Source and Collection

This paper used the data of the 2018 China Family Panel Survey (CFPS) for empirical analysis. By collecting data on individuals, families, and communities, CFPS reflects the development and changes in China’s society, economy, population, health, and education; and provides a foundation of data for academic research and public policy formulation [14]. The CFPS questionnaire consists of four main questionnaire types: community, family, adult, and children. For family members of different natures, there are various types of questionnaires, such as long or short questionnaires, pick-up, and electronic interview questionnaires. In 2018, the CFPS project interviewed 15,000 households and collected data from approximately 44,000 participants. The survey scope covered 31 provinces in China except for Hong Kong, Macao, and Taiwan, with a total effective sample size of 14,218 households and 37,354 individuals. To study healthcare consumption decision-making expenditures at the individual level, according to the research variables required in this paper, a total of 36,735 overall sample datasets were compiled by combining data at the household and individual levels.

### 2.2. Variables

#### 2.2.1. The Dependent Variables

The dependent variables of this paper were health care consumption and consumption decisions. As there was no item of personal health care consumption expenditure in the survey, this study referred to existing research and uses household health care consumption divided by the number of family members to represent residents’ health care consumption expenditure [15]. Health consumption expenditures refer to all consumption expenditures that affect the health of residents [16]. According to the value of health care consumption, a new binary variable consumption decision was generated. If the value of healthcare consumption was 0, the new variable would be assigned a value of 0, indicating that the resident does not participate in healthcare consumption; if the value of healthcare consumption was greater than 0, the new variable would be assigned a value of 1, indicating that the resident participates in healthcare consumption. In this study, whether residents participate in healthcare consumption was used as a dichotomous variable to measure residents’ healthcare consumption decisions.

#### 2.2.2. The Independent Variables

Since the sample of this study covered most of China’s provinces, differences in regional economic development make it difficult to distinguish residents into clear groups of socioeconomic status directly. Drawing on existing research ideas, this study also measured socioeconomic status from three dimensions [7,8]. The core independent variables of this study were income, education, and occupation. The above three indicators are used to evaluate the socioeconomic status of residents comprehensively. This study selected the total personal income from work, including all wages, bonuses, advanced benefits, and in-kind subsidies, and deducted taxes, five social insurance funds, and one housing fund, to measure personal economic level [17]. This study selected the years of education of individuals to measure the educational level of residents. Generally, the longer the residents have been educated, the higher the educational level they have, which can change the behavior and conversation of residents to a certain extent [18]. According to the EGP (Erikson–Goldthorpe–Portocarero schema) occupational class status classification method, this article used EGP occupational class status to measure individual occupational types. In the specific operation, for research needs, this study used Stata software to process the “occupation” variable and simplifies the EGP occupational class into two categories. Namely, “technical occupations” and “labor occupations” [19].

#### 2.2.3. The Mediation Variable and Control Variables

According to existing research findings, residents’ exercise behavior may reduce [20,21] or promote [22] their healthcare consumption. Therefore, the mediating variable of this paper was exercise duration, including the duration of physical education classes, to explore the mediating effect of exercise behavior. In addition, age, health insurance, marital status, urban residence, gender, political status, and family count were introduced as covariates based on previous studies [23,24,25,26]. Marital status, gender, urban status, and political status were all dichotomous variables.

### 2.3. Research Model

The dependent variable consumption decision was a dichotomous variable. The binary probit regression model, as a standard regression and classification algorithm, can measure the uncertainty of classification results by probability, which was helpful in analyzing discrete choice problems. Therefore, the probit regression model was selected to analyze the influence of residents’ socioeconomic status on healthcare consumption participation decision-making, and a benchmark Equation (1) was established.

Due to the large number of 0 values in the sample of healthcare consumption expenditures, the probability distribution of the explained variable became a combination of discrete points and a continuous distribution. If estimated using the OLS method, a consistent estimator may not be obtained. For this reason, this study established a Tobit model suitable for restricted dependent variables and used the maximum likelihood method MLE for estimation. Thus, when the explained variable was healthcare consumption expenditure, the measurement method selected the Tobit model and established a benchmark Equation (2).

(1) $$Probit\left(Consumption\;decision\right)=\beta_{0}+\beta_{1}Income+\beta_{2}Education+\beta_{3}Occupatin+\beta_{4}X+\varepsilon$$

(2) $$Tobit\left(Health\;care\;consumption\right)=\alpha_{0}+\alpha_{1}Income+\alpha_{2}Education+\alpha_{3}Occupation+\alpha_{4}X+\varepsilon$$

To test the mechanism of residents’ physical exercise behavior and the effect of residents’ socioeconomic status on healthcare consumption participation decision-making and expenditure amount, the mediation effect test method was introduced, and mediation effect Equations (3) and (4) were established.

(3) $$Logit\left(Consumption\;decision\right)=\beta_{0}+\beta_{1}Income+\beta_{2}Education+\beta_{3}Occupation+\beta_{4}Exercise\;duration+\beta_{5}X+\varepsilon$$

(4) $$Tobit\left(Health\;care\;consumption\right)=\alpha_{0}+\alpha_{1}Income+\alpha_{2}Education+\alpha_{3}Occupation+\alpha_{4}Exercise\;duration+\alpha_{5}X+\varepsilon$$

*β*_0_ and *α*_0_ are constant terms, *X* was a series of control variables, and *ε* was a random interference term. A detailed explanation of each variable was shown in Table 1. The data analysis used in this study was carried out using Stata software (16, College Station, TX, USA).

### 2.4. Availability of Data

The datasets analyzed in this study are available from the CFPS dataset, http://www.isss.pku.edu.cn/cfps/en/index.htm (accessed on 25 May 2020).

## 3. Results

### 3.1. Descriptive Analysis

The descriptive statistics of the specific variables were shown in Table 1 and Table 2. Among the 37,354 sample data, a total of 5539 residents conducted healthcare consumption in 2018, accounting for only 15.08% of the total number of respondents. Furthermore, the average proportion of residents’ healthcare consumption expenditure in the total consumption expenditure was only 0.5%. This result showed that participants of this survey were less involved in healthcare consumption, and the amount of healthcare consumption only accounted for a small part of household expenditure, which means that attention to it needs to be improved. 

The average was 45 years old, as society as a whole shows an aging trend. In terms of urban variables, the residents participating in the survey were almost evenly distributed in urban and rural areas. This indicated that the data sample of this study would not affect the regression results due to the imbalance between urban and rural areas. In addition, the number of males and females in this study was basically 1:1. There were 4321 individuals with health insurance and 9072 individuals without health insurance, and the insured individuals were only half of the uninsured individuals, indicating that the residents’ awareness of health insurance in the survey group needs to be strengthened.

### 3.2. The Influence of Socioeconomic Status on Healthcare Consumption

Table 3 shows the benchmark regression results on whether to conduct healthcare consumption and healthcare consumption expenditure. Columns (1) and (2) indicated the influence of residents’ socioeconomic status factors on healthcare consumption participation decision-making before and after adding control variables. From the significance level of the marginal effect, after controlling for other variables, residents’ income level and education level had a significant positive impact on healthcare consumption decision-making. That is, the higher the individual’s personal income or education level, the greater the possibility of participating in healthcare consumption. However, occupation had a significant negative effect on the possibility of participating in healthcare consumption. That is, a 1% increase in the resident’s job type score reduces the possibility of a resident participating in medical consumption by 0.005. According to the occupational classification, the higher the occupational score in this study, the more likely the type of work was to be a labor-type job; the lower the score, the more inclined to skilled work. That is, skilled occupations were more likely to consume healthcare. For the control variables, age (0.001) and health insurance (0.052) had a significant positive effect on healthcare consumption decision-making; that is, elderly individuals and residents with health insurance were more motivated to participate in healthcare consumption. Gender (−0.031) and family count (−0.005) had significant negative effects on residents’ healthcare consumption participation and decision-making; that is, women and individuals with fewer family members were more inclined to participate in healthcare consumption. However, the results of the study also found that marital status, urbanization level, and political status had no significant influence on the enthusiasm for participating in healthcare consumption.

Through the Tobit test, the influence of residents’ socioeconomic status on healthcare consumption expenditure was shown in Columns (3) and (4) in Table 3. After controlling for other variables, all socioeconomic status factors had a significant impact on residents’ healthcare consumption expenditure. The influence of the coefficient of income decreased significantly, from 0.045 to 0.029, while the absolute value of the coefficient of education level and occupation type increased significantly. The coefficient increased from 506.085 to 811.149, and the absolute value of the coefficient of occupation type increased from 39.895 to 99.697. Nevertheless, the effects of the three variables on healthcare consumption were still significant at the 1% level. Based on this, Hypotheses 1–3 were verified. For the control variables, age (44.999), medical insurance (1507.781), and urbanization level (137.738) had a significant positive impact on residents’ healthcare consumption expenditure; that is, elderly individuals, medical insurance policyholders, and urban residents had higher healthcare consumption. Gender (−784.933) had a significant negative impact on healthcare consumption expenditure. That is, the amount of women’s healthcare consumption was significantly higher than that of men. However, the influence of marital status and political status on residents’ healthcare consumption expenditure was not significant.

### 3.3. The Mediating Effect of Physical Exercise Duration

To further analyze the potential mechanism of socioeconomic status on residents’ participation in healthcare consumption decision-making and expenditure, Table 4 introduces the duration of residents’ participation in physical exercise based on benchmark regression and conducts a mediating effect test. Table 4 shows that after introducing the intermediary variable, although the absolute value of the marginal effect or coefficient of each variable had decreased compared with Table 3, the effect of the independent variable was still significant. That is, personal income and education level had a significant positive correlation with health consumption participation in decision-making and expenditure amount, while occupational type had a significant negative correlation. The mediating variable also had a significant positive impact on residents’ participation in healthcare consumption and the amount of expenditure.

The research showed that the duration of residents’ physical exercise had a direct impact on the decision-making of residents’ healthcare consumption participation, with a marginal effect of 0.001, which was significant at the 1% level. However, the effect of residents’ physical exercise duration on the amount of residents’ healthcare consumption expenditure was not significant and was only significant at the 10% level. Therefore, Hypothesis 4 was partially verified. The results of the mediation effect test still showed that personal income, education level, and occupation type had indirect effects on residents’ healthcare consumption participation decision-making and the amount of healthcare consumption expenditure through the mediation effect of the duration of residents’ participation in physical exercise. Thus far, Hypothesis 5 has been verified.

### 3.4. Robustness Check

This study referred to previous research methods to avoid the incomplete representation of existing dependent variables on residents’ healthcare consumption and selects the proportion of healthcare consumption expenditure in total expenditure as a replacement dependent variable to measure the rationality of this study [3]. It can be seen from Table 5 that after adding the control variable, the significance of socioeconomic status on the proportion of residents’ healthcare consumption was significantly enhanced, in which residents’ income and education level still showed a significant positive impact, while occupational type had a significant negative impact. The mediating variable also had a positive correlation with the proportion of healthcare consumption. The three types of residents’ socioeconomic status factors still had an indirect impact on healthcare consumption expenditure through the mediating effect of exercise duration; that is, the model used in this study had strong robustness.

## 4. Discussion

Using data from the CFPS survey, this study explored the impact of socioeconomic status on healthcare consumption and the mediating role of physical exercise, with a view to contributing to related research. Using benchmark regression and mediation models, this study produced four valuable findings. First, the level of residents’ income had a significant positive impact on healthcare consumption decisions and expenditures. That is, groups with higher incomes were more likely to consume healthcare, and their healthcare consumption expenditures were significantly higher than those with lower incomes. Hypothesis 1 was supported. As pointed out by Baltagi and Moscone (2010), healthcare consumption was a necessity, and long-term research has found that there is an impact relationship between income and healthcare consumption [27]. Second, education level significantly positively impacted healthcare consumption decision-making and expenditure. That is, the higher the education level of residents, the higher the possibility of participating in healthcare consumption and the higher the corresponding healthcare consumption expenditure. Hypothesis 2 was supported. This was basically consistent with existing research [28], and some scholars had found that parents’ education level also had a positive impact on children’s healthcare consumption [29,30]. Third, the occupational type had a significant positive impact on healthcare consumption decisions and expenditures; that is, residents with skilled jobs were more likely to participate in healthcare consumption and had higher healthcare consumption expenditures. Hypothesis 3 was supported. As a Korean study found, skilled workers tended to spend more on healthcare than workers in simple labor [31]. Fourth, exercise duration was significantly positively correlated with healthcare consumption, and physical exercise also played a mediating role in the relationship between socioeconomic status and residents’ healthcare consumption. Hypotheses 4 and 5 were supported. Some studies had linked physical activity with healthcare consumption, but few studies had pointed out the mediating effect of physical activity [32]. Thus, this study contributed to a better understanding of the relationship between socioeconomic status, physical activity, and healthcare consumption.

Some studies have shown the influence of socioeconomic status on healthcare consumption [17,33,34]. Some researchers believe that socioeconomic status may be a decisive factor affecting individual consumption levels and living habits [35]. Similar to previous findings, this study also revealed a significant influence of socioeconomic status on healthcare consumption decisions and healthcare consumption expenditures. When people were from a higher socioeconomic status, they were less limited in terms of food and clothing, tended to improve their quality of life, and thus paid more attention to physical health. Therefore, healthcare consumption, including sports consumption, had gradually become popular among high-class residents. For example, people with higher incomes were more likely to have access to higher-end healthcare products because high-end products were usually associated with higher selling prices, so the level of healthcare consumption of such residents was also relatively high [36]. Likewise, people with higher education levels and those with skill-oriented occupations generally had higher-end consumer demand, thereby increasing the level of healthcare consumption [37]. It can be seen that the improvement of residents’ socioeconomic status would be accompanied by an increase in healthcare consumption expenditure. Conversely, residents of lower socioeconomic status paid more attention to basic consumption, such as food and clothing, resulting in less disposable money in the field of healthcare consumption. 

In addition, some studies have pointed out that there was a negative impact on socioeconomic status and healthcare consumption. As Loef et al. (2021) found in a cross-sectional study in the Netherlands, groups with lower education and income levels had higher total healthcare expenditures than those with higher socioeconomic status [38]. This had certain commonalities with the findings of this study; that is, socioeconomic status was associated with healthcare consumption but in the opposite direction. The possible reason for this was that social welfare policies in developed countries were generally better developed, so groups with lower socioeconomic bases could afford more healthcare consumption through welfare policies [39,40,41]. In contrast, in China, most lower socioeconomic groups, especially elderly individuals, usually consciously choose to avoid the occasions requiring healthcare consumption. Because of the influence of Chinese culture, “thrift is a virtue,” and healthcare consumption was usually the middle to high level of consumer demand in Maslow’s hierarchy of needs. Therefore, in China, lower socioeconomic groups may have relatively fewer healthcare consumption needs.

Another finding of this study was that the duration of residents’ physical activity had a significant effect on healthcare consumption, which was consistent with existing research. Numerous studies have shown that there was a positive relationship between physical activity and healthcare consumption [13,42]. Most of the studies were carried out from the aspects of physical exercise venues, sports type, time, intensity, and so on. Whether it was the consumption of nutritious food to help residents improve their exercise effect, the auxiliary technology equipment to ensure the intensity of exercise, the sports venues that provided professional facilities, or the social sports instructors who provided professional training guidance, they were all related to higher-level consumption [43]. However, there were also studies pointing to an inverse relationship between exercise and healthcare consumption. For example, a study on the elderly in Japan found that regular stepping exercise could reduce the healthcare expenditure of the elderly [44]. There were still similarities with this study; that is, exercise could cultivate residents’ awareness of sports and improve their physical fitness. However, in China, the increase in the sports injury rate caused by factors such as a lack of scientific guidance has also become an important reason for the increase in healthcare consumption expenditures due to exercise [45]. In general, the results of this study further proved that physical exercise could help cultivate residents’ awareness of fitness and healthcare and gradually increase their demand for healthcare consumption.

There were few existing studies on the mechanism of physical exercise in the relationship between socioeconomic status and healthcare consumption. However, the present study found that a mediation effect model supports a mediating role of physical activity in the relationship between socioeconomic status and healthcare consumption. A possible explanation was that physical exercise, as a form of socialization, was generally popular among people of higher socioeconomic status, such as socializing while golfing [46]. Abundant forms of physical exercise, paired with more expensive equipment and sportswear, had resulted in higher healthcare consumption expenditures by people from a higher social stratum. The purpose of their participation in sports may not only be health but also work or socially related, such as managing a project or making friends while exercising, thereby contributing to personal development [47]. That is to say, the socioeconomic status of residents can directly affect healthcare consumption through physical exercise.

## 5. Strengths and Limitations

This research had certain theoretical and practical significance. On the one hand, this study found that social status had an important impact on healthcare consumption based on survey data at the micro-level in China. On the other hand, existing studies have not explored the internal mechanism in detail. This study introduced physical exercise variables as mediating variables to explain the impact of socioeconomic status on healthcare consumption to a certain extent. In addition, this study provided an evidence base for policy formulation to promote sports consumption further and regulate medical consumption. This study highlighted the importance of physical activity in healthcare consumption. Therefore, in the process of formulating relevant policies, such as “national fitness,” attention should be paid to the relationship between physical exercise and consumption. Policies should reflect the realization that the sports industry will become a pillar industry of China’s national economy.

However, this study still had some limitations. First, the data related to healthcare consumption were the average data of each household used, which may have certain personal errors. Second, although the mediation model can test the relationship between variables, the accuracy of the results was not perfect because this study only used cross-sectional data. This study used a cross-sectional design rather than a longitudinal design, so a causal relationship between socioeconomic status and healthcare consumption was unable to be established. Therefore, this study can only explain the relationship between income level, education level, occupation type, and healthcare consumption while also exploring the mediating mechanism through exercise frequency. Third, due to the limitation of data variables, this study had not been tested for endogeneity, which may threaten internal validity. There may still be confounding factors that cannot be observed in this study, such as China’s economic divisions, including the four major economic regions of eastern, western, central, and north-eastern China. Because the level of economic development varies greatly between regions, it may affect the relationship between socioeconomic status and healthcare consumption. In addition, in the dichotomous variables that measure healthcare consumption decisions, there was a large amount of data that is 0, which may hide heterogeneous information.

Therefore, for future research, it is recommended to use longitudinal data combined with regional differences in China to conduct an endogeneity test to improve the reliability of the results. In addition, to narrow socioeconomic disparities and promote the growth of healthcare consumption, this study also makes several policy recommendations. First, to increase investment in scientific research and provide diversified healthcare products and services. The scientific and technological research and development of products should be strengthened, the healthcare needs of different groups should be met, and the differences in healthcare consumption caused by inequality of socioeconomic status should be reduced. Second, fitness publicity should be increased and people should be encouraged to participate in sports. Full play should be given to the mediating role of exercise between socioeconomic status and healthcare consumption. By cultivating residents’ awareness of exercise, the purpose of promoting healthcare consumption by various groups of people is achieved.

## 6. Conclusions

This study examined the impact of residents’ socioeconomic status on healthcare consumption decisions and expenditures and focused on the mediating role of physical activity duration. The results showed that socioeconomic status is significantly positively correlated with healthcare consumption decisions and healthcare consumption expenditures. At the same time, the longer residents participate in sports, the higher the possibility of participating in healthcare consumption. In addition, physical activity duration had a significant mediating effect between socioeconomic status and healthcare consumption. This suggested that residents with higher socioeconomic status tend to engage in physical activity more frequently and for longer. Based on participating in sports for a long time, residents tended to create more spending on healthcare.

These findings enhanced our understanding of the relationships and mediating mechanisms among physical activity, socioeconomic status, and healthcare consumption. Therefore, it was strongly recommended that the government pay attention to the phenomenon of increasing social stratification and the widening gap between the rich and the poor, and thus provide diversified public fitness services inclusive of the whole population, realize the fair distribution of resources, increase the frequency and time of residents’ exercise, and improve the healthcare consumption level of different socioeconomic statuses.

## Figures and Tables

**Table 1:** Descriptive statistical results of continuous variables.

Variable	Description	Number	Mean	SD
**The dependent variables**				
Healthcare consumption	Healthcare consumption expenditure (measured in Chinese yuan)	36,735	443.155	2817.426
Proportion of healthcare consumption	Proportion of healthcare consumption in total consumption expenditure	36,639	0.005	0.037
**The independent variables**				
Income	Gross income from work (measured in Chinese yuan)	36,735	11,732.730	25,863.510
Education	Education level (measured in years)	18,400	4.020	2.319
**Control variables**				
Age	Age	36,735	44.884	19.365
Family count	Number of family members	34,696	3.456	3.519
**The mediating variable**				
Exercise duration	Weekly workout duration (measured in hours)	32,769	0.115	11.160

**Table 2:** Descriptive statistical results of category variables.

Variable	Description	Frequency	%
**The dependent variables**			
Consumption decision	Whether or not to consume healthcare (yes = 1, no = 0)		
	Yes	5539	15.08
	No	31,196	84.92
**The independent variables**			
Occupation	Type of jobs (technical occupations = 1, labor occupations = 2)		
	Technical occupations	15,851	64.58
	Labor occupations	8696	35.42
**Control variables**			
Health insurance	Whether the resident has health insurance (yes = 1, no = 0)		
	Yes	4321	32.26
	No	9072	67.74
Marital status	Unmarried = 1, married = 2		
	Unmarried	3916	11.95
	Married	28,853	88.05
Urban	Urban = 1, rural = 0		
	Urban	18,147	49.40
	Rural	18,588	50.60
Gender	Male = 1, female = 0		
	Male	18,283	49.77
	Female	18,452	50.23
Political status	Whether or not a member of the Communist Party of China (yes = 1, no = 0)		
	Yes	2748	7.48
	No	33,987	92.52

**Table 3:** The results of the impact of socioeconomic status on residents’ health care consumption decisions and expenditures.

Variables	Consumption Decision		Healthcare Consumption	
	(1) Probit	(2) Probit	(3) Tobit	(4) Tobit
Income	1.45 × 10^−6^ ***	8.44 × 10^−7^ ***	0.045 ***	0.029 ***
	(9.13 × 10^−8^)	(1.22 × 10^−7^)	(0.003)	(0.003)
Education	0.012 ***	0.030 ***	506.085 ***	811.149 ***
	(0.001)	(0.003)	(50.819)	(80.727)
Occupation	−0.004	−0.005 *	−39.895 ***	−99.697 ***
	(0.005)	(0.007)	(36.371)	(37.347)
Age		0.001 ***		44.999 ***
		(0.001)		(15.556)
Health insurance		0.052 ***		1507.781 ***
		(0.010)		(280.043)
Marital status		0.002		68.046
		(0.007)		(202.071)
Urban		0.045 ***		137.738 *
		(0.008)		(76.646)
Gender		−0.031 ***		−784.993 ***
		(0.009)		(242.714)
Political status		0.021		383.970
		(0.014)		(408.001)
Family count		−0.005 ***		−76.618
		(0.002)		(53.513)
_cons	−1.310 ***	−1.665 ***	−11,676.200 ***	−13,540.360 ***
	(0.024)	(0.093)	(326.508)	(796.715)
Prob > chi2	0.000	0.000	0.000	0.000
Pseudo R^2^	0.034	0.057	0.009	0.010
*N*	13,091	8972	13,091	8972

Note: (1) * *p* < 0.1. ** *p* < 0.05. *** *p* < 0.01. (2) Robust standard error for *t*-statistics in parentheses. (3) Columns (1) and (2) report the marginal effect.

**Table 4:** Mediating effect results from residents’ physical exercise duration.

Variables	Consumption Decision	Healthcare Consumption
	(1) Probit	(2) Tobit
Income	8.51 × 10^−7^ ***	0.029 ***
	(1.22 × 10^−7^)	(0.003)
Education	0.030 ***	803.423 ***
	(0.003)	(80.914)
Occupation	−0.005 *	−98.576 ***
	(0.007)	(37.365)
Exercise duration	0.001 ***	21.678 *
	(0.0004)	(12.970)
Age	0.001 **	43.591 ***
	(0.0005)	(15.583)
Health insurance	0.051 ***	1486.469 ***
	(0.010)	(280.421)
Marital status	0.002	77.570
	(0.007)	(202.116)
Urban	0.045	134.231 *
	(0.008)	(76.684)
Gender	−0.032 ***	−811.954 ***
	(0.009)	(243.385)
Political status	0.020	367.300
	(0.014)	(408.323)
Family count	−0.005 ***	−76.047
	(0.002)	(53.525)
_cons	−1.634 ***	−13,430.980 ***
	(0.093)	(799.237)
Prob > chi2	0.000	0.000
Pseudo R^2^	0.058	0.010
*N*	8972	8972

Note: (1) * *p* < 0.1. ** *p* < 0.05. *** *p* < 0.01. (2) Robust standard error for *t*-statistics in parentheses. (3) Column (1) reports the marginal effect.

**Table 5:** Robustness test results.

Variables	Proportion of Healthcare Consumption	
	(1)	(2)
Income	4.01 × 10^−7^ ***	1.88 × 10^−7^ ***
	(3.36 × 10^−8^)	(3.45 × 10^−8^)
Education	0.005 ***	0.008 ***
	(0.001)	(0.001)
Occupation	0.00004	−0.001 *
	(0.0004)	(0.0004)
Exercise duration		0.0003 *
		(0.0002)
Control variables		Controlled
_cons	−0.131 ***	−0.127 ***
	(0.004)	(0.008)
Prob > chi2	0.000	0.000
Pseudo R^2^	0.155	0.396
*N*	13,068	8955

Note: (1) * *p* < 0.1. ** *p* < 0.05. *** *p* < 0.01. (2) Robust standard error for *t*-statistics in parentheses.

## Data Availability

The data can be obtained at: http://www.isss.pku.edu.cn/cfps/en/index.htm (accessed on 25 May 2020).
